# Alleviation of chromium toxicity in mung bean (*Vigna radiata* L.) using salicylic acid and *Azospirillum brasilense*

**DOI:** 10.1186/s12870-023-04528-w

**Published:** 2023-11-03

**Authors:** Hafiz Haider Ali, Maimoona Ilyas, Muhammad Saqlain Zaheer, Akhtar Hameed, Kamran Ikram, Waqas ud Din Khan, Rashid Iqbal, Tahir Hussain Awan, Muhammad Rizwan, Abd El-Zaher M. A. Mustafa, Mohamed Soliman Elshikh

**Affiliations:** 1 Department of Agriculture, Government College University Lahore, Lahore, 54000 Pakistan; 2 Sustainable Development Study Center (SDSC), Government College University Lahore, Lahore, 54000 Pakistan; 3 Department of Agricultural Engineering, Khwaja Fareed University of Engineering and Information Technology, Rahim Yar Khan, 64200 Pakistan; 4 Institute of Plant Protection, MNS University of Agriculture, Multan, 61000 Pakistan; 5 Department of Agronomy, Faculty of Agriculture and Environment, The Islamia University of Bahawalpur, Bahawalpur, 63100 Pakistan; 6 Department of Agronomy, Rice Research Institute, Kala Shah Kaku, Lahore, 54000 Pakistan; 7 Department of Plant Nutrition, Institute of Crop Science and Resource Conservation (INRES), University of Bonn, Bonn, 53115 Germany; 8 Department of Botany and Microbiology, College of Science, King Saud University, P.O. 2455, Riyadh, 11451 Saudi Arabia

**Keywords:** *Azospirilium brasilense*, Chromium toxicity, Mung bean, Plant growth, Salicylic acid

## Abstract

**Background:**

Chromium (Cr) contamination in soil poses a serious hazard because it hinders plant growth, which eventually reduces crop yield and raises the possibility of a food shortage. Cr’s harmful effects interfere with crucial plant functions like photosynthesis and respiration, reducing energy output, causing oxidative stress, and interfering with nutrient intake. In this study, the negative effects of Cr on mung beans are examined, as well as investigate the effectiveness of *Azospirillum brasilense* and salicylic acid in reducing Cr-induced stress.

**Results:**

We investigated how different Cr levels (200, 300, and 400 mg/kg soil) affected the growth of mung bean seedlings with the use of *Azospirillum brasilense* and salicylic acid. Experiment was conducted with randomized complete block design with 13 treatments having three replications. Significant growth retardation was caused by Cr, as were important factors like shoot and root length, plant height, dry weight, and chlorophyll content significantly reduced. 37.15% plant height, 71.85% root length, 57.09% chlorophyll contents, 82.34% crop growth rate was decreased when Cr toxicity was @ 50 µM but this decrease was remain 27.80%, 44.70%, 38.97% and 63.42%, respectively when applied *A. brasilense* and Salicylic acid in combine form. Use of *Azospirillum brasilense* and salicylic acid significantly increased mung bean seedling growth (49%) and contributed to reducing the toxic effect of Cr stress (34% and 14% in plant height, respectively) due to their beneficial properties in promoting plant growth.

**Conclusions:**

Mung bean seedlings are severely damaged by Cr contamination, which limits their growth and physiological characteristics. Using *Azospirillum brasilense* and salicylic acid together appears to be a viable way to combat stress brought on by Cr and promote general plant growth. Greater nutrient intake, increased antioxidant enzyme activity, and greater root growth are examples of synergistic effects. This strategy has the ability to reduce oxidative stress brought on by chromium, enhancing plant resistance to adverse circumstances. The study offers new perspectives on sustainable practices that hold potential for increasing agricultural output and guaranteeing food security.

## Introduction

The sixth most common transition metal and the seventh most prevalent element in the earth’s crust is chromium (Cr) [1]. Due to the weathering of the earth’s crust and the deposition of waste from industrial operations, including the chemical (leather, pigments, electroplating, and other industries) and metallurgical (mostly steel and metal) sectors, it is present in the environment [2]. Environmental conditions severally effect on the growth and development of the plants [3, 4]. Most environmental matrices (air, water, and soil) contain this element, which has recently expanded dramatically in aquatic and terrestrial ecosystems [5]. Cr is widely used in industries like steel, leather, and textiles. The common effects are degraded pigment status, inhibition of seed germination, nutrient imbalance, antioxidant enzymes, changes in chloroplast and membrane ultrastructure, and water stress in plants [6]. Industrial runoff is a main source of chromium contamination in the agricultural fields. Sites close to wastewater release sites and industrial areas are particularly vulnerable to chromium buildup, which could harm crops and the environment [2]. The addition of heavy metals to the environment has increased alarmingly all around the world over the last couple of decades and is an important cause of a reduction in plant growth, especially in developing countries like Pakistan.

Mung bean (*Vigna radiate* L.) also known as green gram in Asia and also a widely used legume (pulse) crop for providing protein throughout the region [7]. Due to their abundance in protein, certain minerals, vitamins, and calories, pulse crops are considered nutritional crops for the consumption of human beings, and mung bean can be eaten in a variety of ways boiled, fried, sprouted, and in powdered form, and most nutrient dense form of mung bean is mung bean sprout, which has greater than 200% more protein than other consumable forms. 100-gram mung bean sprouts have a nutritional content of 7 g of protein, 18 g of carbohydrate portion, 0.026 g of sodium, 24 gram of fat, 0.0029gram of calcium, and 103.5 calories and other significant vitamins [8]. Mung bean is the 2nd largest cultivated crop in Pakistan after chickpea. It is planted as a cash crop in summer and spring season. Mung bean cultivated on 0.25 million hectares of land with the annual production of 178 tones and averaged yield of this crop is around 515 kg/ha [9].

Inoculating plants with Azospirillum, which principally stimulates plant root development, may help to boost and stabilize crop yield. Although they have been carried out on various crops and in various regions, evaluations of Azospirillum’s effectiveness under current cultivation practices and at typical environmental conditions are rare [10]. Under field conditions, *Azospirillum brasilense* can colonize hundreds of plant species and significantly enhance their growth, development, and efficiency. The most researched observed the mechanism for Azospirillum to promote plant growth in inoculated plants, in addition to nitrogen fixation, has been linked to its capacity to produce a number of phytohormones, primarily auxins and particularly indole-3-acetic acid [11], abscisic acid (ABA), polyamines, ethylene, and nitric oxide [12]. Under controlled agronomic conditions, *Azospirillum brasilense* can colonize the soil rhizosphere and in the internal tissues of many plants that directly enhancing their proliferation, development, and yield. Azospirillum has both direct and indirect effects on plant development. Increases in the bioavailability of nutrients for plants (such as nitrogen, phosphorus, iron, and potassium) or the creation of enzymes and plant growth regulators (phytohormones) are examples of direct methods. Plant resistance to infections and tolerance to abiotic stress are two examples of indirect processes [13]. Under abiotic stress conditions, SA’s ability to restore growth is correlated with its impact on physiological aspects of the plant, including water content, nutrient uptake, synthesis of chlorophyll pigments, growth, stomatal regulation, suppression of ethylene biosynthesis, hormonal profile regulation, and protein kinas synthesis [14]. In extremely modest amounts, SA is an endogenous growth regulator that occurs naturally in plants [15]. There is a need to assess the impact of metals such chromium on the crop production and the impact on important cash crops of Pakistan. The impact of chromium on the mung bean’s germination and growth efficiency is poorly understood. The current study planned to evaluate the impact of differing chromium concentrations on seed germination and plant growth performance. We hypothesized that the combine application of *A. brasilense* and SA might have the positive impact on mung bean under Cr toxicity and can improve the plant growth. This study explores a novel approach to enhance plant tolerance to heavy metal stress by using combine application of *A. brasilense* and a natural plant hormone (CA), providing valuable insights into sustainable agricultural practices for managing metal-contaminated soils.

## Materials and methods

### Experimental design

An approved mung bean variety “PRI Mung-2018” seeds were obtained from the Pulses Research Institute, Faisalabad for research. Six mung bean seeds were planted in a clay pot at a depth of 10 cm, containing 2 kg of that soil that had been carefully rinsed and dried to examine the effects of *Azospirillum brasilense* and salicylic acid on mung bean plants under chromium-contaminated soil. To create the chromium toxicity, Soil was contaminated with the chromium compound {potassium dichromate (K2Cr2O7)} @200, 300, and 400 mg/kg of the soil as per the treatments. Cr was applied in the soil 5 days before the sowing and uniformly mixed in each pot. Soil sampling and analysis are represented in Table 1. *Azospirillum brasilense* (FCBP-SB0025) (B3) pure culture was obtained from the Fungal Culture Bank of Pakistan (FCBP) at the Faculty of Agriculture Sciences, University of Punjab, Lahore. A total of 39 plastic pots were arranged in three replications of 13 treatment combinations using randomized block design (RBD) in a greenhouse at the Botanical Garden, Government College, University of Lahore. The greenhouse environment includes controlled temperature (often 20–30 °C), relative humidity (commonly 50–70%), photoperiod (12–16 h of light), and additional lighting to promote optimal plant growth, producing a space suited for study and experimentation. The pot trial was performed from March 28 to April 27, 2022, in a greenhouse.

**Table 1:** Characteristics of sandy loam soil used in pot experiment

Parameters	Percentage
Soil texture	Clay loam
Sand	38%
Silt	32%
Clay	36%
Organic matter	0.86%
pH	7.48
EC	1.37 dSm^−1^
CEC	5.96 cmol Kg^−1^

### Parameters studied

#### Plant height, shoot root length, fresh and dry weight

Measuring the vertical plant height from the base to the tallest shoot tip is the first step in gathering information on “Plant height, shoot root length, fresh and dry weight” in mung bean plants. Measure the longest root and main stem, respectively, to determine the length of the shoot. Immediately weigh the entire uprooted plant to determine its fresh weight. Dry the plant in an oven to determine dry weight, and then weigh it once it has reached a steady weight. The plant dry weight was determined by drying the 3 tallest plants from each replicate for each concentration, were dried in a hot air oven at 60 °C for 48 h. Repeat the procedure for numerous plants in each treatment, using averages to improve data accuracy. All the relevant data was collected after 1 month of the seed germination.

### Crop growth rate (CGR)

Crop growth rate was noticed by the following formula as reported by Karimi and Siddique [16].

$$\mathrm{CGR}\;\;=\;\left({\mathrm W}_2\;-\;{\mathrm W}_1\;\right)\;/\;\left({\mathrm t}_2\;-\;{\mathrm t}_1\;\right)$$

Where:

W_2_: Final dry weight of the crop (grams per square meter)

W_1_: Initial dry weight of the crop (grams per square meter)

t_2_: Final time (days)

t_1_: Initial time (days)

### Chlorophyll contents and relative water contents

Chlorophyll contents (a, b, and total) were also estimated by using the procedure described by Arnon [17]. Relative water content in the leaf was noticed by Barrand Weatherley’s equation [18] as follows,

$$\mathrm{RWC}\;(\%)\;=\;\lbrack(\mathrm{FW}\;-\;\mathrm{DW})\;/\;(\mathrm{TW}\;-\;\mathrm{DW})\rbrack\;\times\;100.$$

5 g of fresh leaves were acquired, and they were mashed in a pestle and mortar with 80% acetone to detect the chlorophyll a and b. After being ground, each sample was boosted in volume by 10 mL using acetone before being centrifuged for five minutes at 4000 rpm. This sample’s absorbance was determined using a UV/visible spectrophotometer (Spectro scan 80D, Kyoto, Japan) at 663 and 645 nm. The following formulas were used to determine the concentrations of chlorophyll a, chlorophyll b, and total chlorophyll as reported by Du et al. [19]:

$$\mathrm{The}\;\mathrm{formula}\;\mathrm{for}\;\mathrm{chlorophyll}\;\mathrm a\;(\mathrm{Chl}\;\mathrm a)\;\mathrm{is}\;\mathrm{Chl}\;\mathrm a\;(\mathrm{mg}/\mathrm{ml})\;=\;12.7\;\ast\;(\mathrm A665)\;-\;2.69\;\ast\;(\mathrm A652).$$

$$\mathrm{The}\;\mathrm{formula}\;\mathrm{for}\;\mathrm{chlorophyll}\;\mathrm b\;(\mathrm{Chl}\;\mathrm b)\;\mathrm{is}\;\mathrm{Chl}\;\mathrm b\;(\mathrm{mg}/\mathrm{ml})\;=\;22.9\;\ast\;(\mathrm A652)\;-\;4.68\;\ast\;(\mathrm A665).$$

$$\mathrm{Total}\;\mathrm{Chlorophyll}\;(\mathrm{Mg}/\mathrm{ml})\;=\;\mathrm{Total}\;\mathrm{Chl}\;(\mathrm{mg}/\mathrm{ml})\;+\;\mathrm{Total}\;\mathrm{Chl}\;(\mathrm{Mg}/\mathrm{ml})$$

Where A665 and A652 are the absorbance figures discovered through spectrophotometric analysis at the appropriate wavelengths.

### Enzymatic activities

Harvesting plant tissue and making a homogenised extract in a cold buffer solution are the first steps in measuring the activity of various enzymes in mung bean plants, such as catalase (CAT), ascorbate peroxidase (APX), peroxidase (POD), and superoxide dismutase (SOD). 240 nm hydrogen peroxide (H_2_O_2_) breakdowns should be observed for catalase, and enzyme activity should be calculated based on the change in absorbance. By detecting the ascorbate’s reduction by H_2_O_2_ at 290 nm, one can measure the activity of ascorbate peroxidase. Track the oxidation of the substrate (such as guaiacol) in the presence of H_2_O_2_ to determine the activity of the peroxidase. Determine the superoxide dismutase’s ability to prevent the photoreduction of nitro blue tetrazolium (NBT) at 560 nm and express activity in accordance with that finding. Ensuring correct reporting of enzyme activity per gramme of fresh or dry plant tissue weight. Ascorbate peroxidase (APX), catalase (CAT), Peroxidase (POD), and superoxide dismutase (SOD) activities were noticed according to the Nakano and Asada [20], Vanacker et al. [21], Ghanati et al. [22], and Beyer and Fridovich [23], respectively. All local, national or international guidelines and legislation were adhered to for the use of plants in this study.

### Statistical analysis

All data was analyzed at a 95% probability level by using Fisher’s test, and least significant difference (LSD) with the use of Statistix 8.1 computer software.

## Results

### Effect of *Azospirillum brasilense* and salicylic acid on growth related parameters of mung bean under chromium toxicity

Chromium (Cr) toxicity showed the significant negative effect on the mung bean seedlings Highest plant height, shoot length, root length, shoot fresh weight, root fresh weight, shoot dry weight and root dry weight was noticed in T_1_ under control condition when no Cr stress was applied having no soil amendment. Highest plant height (32.22 cm), shoot length (21.07 cm), root length (11.05 cm), shoot fresh weight (16.64 g), root fresh weight (1.01 g), shoot dry weight (9.82 g) and root dry weight (0.65 g) was noticed in T_1_ under control condition when no Cr stress was applied having no soil amendment followed by T_5_ (plant height: 30.18 cm, shoot length: 19.43 cm, root length: 9.02 cm, shoot fresh weight: 13.41 g, root fresh weight: 0.96 g, shoot dry weight: 8.43 g and root dry weight: 0.60 g, respectively) when Cr toxicity was 200 mg/kg of soil having seed inoculation of *A. brasilense* and foliar application of SA. Lowest results were noticed in T_10_ (plant height: 20.25 cm, shoot length: 11.13 cm, root length: 3.11 cm, shoot fresh weight: 6.12 g, root fresh weight: 0.68 g, shoot dry weight: 3.02 g and root dry weight: 0.13 g, respectively) when Cr toxicity was 400 mg/kg soil having no amendment (Table 2).

**Table 2:** Effect of salicylic acid (SA) and A. *brasilense* on plant height, shoot and root length, root shoot dry and fresh weight and chlorophyll content of mung bean plant under Cromium (Cr) toxicity

**Treatments**	**Plant Height (cm)**	**Shoot Length (cm)**	**Root Length (cm)**	**Shoot Fresh Weight (g)**	**Root Fresh Weight (g)**	**Shoot Dry Weight (g)**	**Root Dry Weight (g)**	**Chlorophyll** **(mg ml**^**−1**^**)**		**Total chlorophyll** **(mg ml**^**−1**^**)**
								a	b	
T_1_ = Control	32.22 a	21.07 a	11.05 a	15.64 a	1.01 a	9.82 a	0.65 a	2.15 a	1.16 a	3.31 a
T_2_ = Cr @200 mg/kg soil	27.18 e	15.07 e	6.74 e	10.01 e	0.80 e	5.92 e	0.47 e	1.70 e	0.94 e	2.64 e
T_3_ = Cr@200 mg/kg soil + *A. brasilense*	29.43 c	18.15 c	8.11 c	12.31 c	0.94 c	7.22 c	0.57 c	1.97 c	1.06 c	3.03 c
T_4_ = Cr@200 mg/kg soil + Salicylic acid	28.22 d	17.33 d	7.41 d	11.28 d	0.92 d	6.52 d	0.52 d	1.87 d	0.98 d	2.86 d
T_5_ = Cr@200 mg/kg soil + *A. brasilense* + Salicylic acid	30.18 b	19.43 b	9.02 b	13.41 b	0.96 b	8.43 b	0.60 b	2.09 b	1.12 b	3.21 b
T_6_ = Cr @300 mg/kg soil	24.35 i	13.12 i	4.60 i	9.02 i	0.72 i	4.53 i	0.31 i	1.32 i	0.81 h	2.13 i
T_7_ = Cr@300 mg/kg soil + *A. brasilense*	26.27 g	16.32 g	6.27 g	11.10 g	0.84 g	5.11 g	0.39 g	1.52 g	0.89 f	2.42 g
T_8_ = Cr@300 mg/kg soil + Salicylic acid	25.15 h	15.12 h	5.66 h	10.2 h	0.82 h	4.84 h	0.36 h	1.48 h	0.86 g	2.34 h
T_9_ = Cr@300 mg/kg soil + *A. brasilense* + Salicylic acid	27.67 f	17.12 f	7.28 f	12.08 f	0.87 f	5.43 f	0.43 f	1.62 f	0.92e,f	2.54 f
T_10_ = Cr@400 mg/kg soil	20.25 m	11.13 m	3.11 m	6.12 m	0.68 m	3.02 m	0.13 m	0.81 m	0.61 l	1.42 m
T_11_ = Cr@400 mg/kg soil + *A. brasilense*	22.31 k	15.18 k	5.3 k	7.21 k	0.75 k	3.87 k	0.23 k	1.01 k	0.72 j	1.73 k
T_12_ = Cr@400 mg/kg soil + Salicylic acid	21.46 l	14.15 l	4.11 l	8.11 l	0.70 l	3.59 l	0.19 l	0.94 l	0.68 k	1.62 l
T_13_ = Cr@400 mg/kg soil + *A. brasilense* + Salicylic acid	23.26 j	16.13 j	6.11 j	9.42 j	0.77 j	4.11 j	0.27 j	1.26 j	0.76 i	2.02 j

### Effect of *Azospirillum brasilense* and salicylic acid on chlorophyll contents of mung bean under chromium toxicity

Data regarding the chlorophyll contents shows that all studied treatments significantly effected on the chlorophyll contents (Table 2). Highest chlorophyll a (2.15 mg/ml), b (1.16 mg/ml) and total chlorophyll contents (3.31 mg/ml) were noticed in T_1_ under control conditions having no toxicity followed by T_5_ (chlorophyll a: 2.09 mg/ml, chlorophyll b:1.12 mg/ml and total chlorophyll: 3.21 mg/ml, respectively) when Cr toxicity was @200 mg/kg of soil with the combine application of *A. brasilense* and SA. Lowest results (chlorophyll a: 0.81 mg/ml, chlorophyll b: 061 mg/ml and total chlorophyll: 1.42 mg/ml, respectively) were noticed in T10 when Cr toxicity was @ 400 mg/kg of soil having no amendment.

### Effect of *Azospirillum brasilense* and salicylic acid on enzymatic activities of mung bean under chromium toxicity

Enzymatic activities of mung bean were significantly affected by the application of *Azospirillum brasilense* and salicylic acid under chromium toxicity (Table 3). Higher negative effects were noticed with the increase of Cr toxicity and combine application of *A. brasilense* and SA is effective to control their negative effect. Highest CAT (Catalase), APX (Ascorbate peroxidase), POD (peroxidase) and SOD (superoxide dismutase) activities was noticed in T_1_ (CAT: 1.32 units/mg of protein, APX: 4.52 units/mg of protein, POD: 0.82 units/mg of protein, SOD: 4.01 units/mg of protein) followed by T_5_ (CAT: 1.21 units/mg of protein, APX: 4.20 units/mg of protein, POD: 0.71 units/mg of protein, SOD: 3.81 units/mg of protein) and lowest results were seen in T_10_ (CAT: 0.68 units/mg of protein, APX: 1.22 units/mg of protein, POD: 0.08 units/mg of protein, SOD: 2.62 units/mg of protein).

**Table 3:** Effect of salicylic acid (SA) and A. *brasilense* on catalase (CAT), ascorbate peroxidase, (APX), peroxidase (POD) and superoxide dismutase (SOD) of mung bean plant under Cromium (Cr) toxicity

Treatments	CAT	APX	POD	SOD
	units/mg of protein			
T_1_ = Control	1.32 a	4.52 a	0.82 a	4.01 a
T_2_ = Cr@200 mg/kg soil	0.93 e	3.32 e	0.47 e	3.61 e
T_3_= Cr@200 mg/kg soil + *A. brasilense*	1.09 c	3.90 c	0.64 c	3.76 c
T_4_ = Cr@200 mg/kg soil + Salicylic acid	0.97 d	3.60 d	0.58 d	3.68 d
T_5_= Cr@200 mg/kg soil + *A. brasilense* + Salicylic acid	1.21 b	4.20 b	0.71 b	3.81 b
T_6_= Cr@300 mg/kg soil	0.82 i	2.21 i	0.21 i	3.23 i
T_7_= Cr@300 mg/kg soil + *A. brasilense*	0.87 g	2.80 g	0.31 g	3.43 g
T_8_ = Cr@300 mg/kg soil + Salicylic acid	0.84 h	2.50 h	0.26 h	3.31 h
T_9_= Cr@300 mg/kg soil + *A. brasilense* + Salicylic acid	0.92 f	3.07 f	0.39 f	3.51 f
T_10_= Cr@400 mg/kg soil	0.68 m	1.22 m	0.08 m	2.62 m
T_11_= Cr@400 mg/kg soil + *A. brasilense*	0.76 k	1.80 k	0.14 k	2.94 k
T_12_ = Cr@400 mg/kg soil + Salicylic acid	0.72 l	1.51 l	0.11 l	2.75 l
T_13_= Cr@400 mg/kg soil + *A. brasilense* + Salicylic acid	0.79 j	2.07 j	0.18 j	3.10 j

Enzymatic activities of mung bean were significantly affected by the application of *Azospirillum brasilense* and salicylic acid under chromium toxicity (Table 3). Higher negative effects were noticed with the increase of Cr toxicity and combine application of *A. brasilense* and SA is effective to control their negative effect. Highest CAT (Catalase), APX (Ascorbate peroxidase), POD (peroxidase) and SOD (superoxide dismutase) activities was noticed in T_1_ (CAT: 1.32 units/mg of protein, APX: 4.52 units/mg of protein, POD: 0.82 units/mg of protein, SOD: 4.01 units/mg of protein) followed by T_5_ (CAT: 1.21 units/mg of protein, APX: 4.20 units/mg of protein, POD: 0.71 units/mg of protein, SOD: 3.81 units/mg of protein) and lowest results were seen in T_10_ (CAT: 0.68 units/mg of protein, APX: 1.22 units/mg of protein, POD: 0.08 units/mg of protein, SOD: 2.62 units/mg of protein).

### Effect of *Azospirillum brasilense* and salicylic acid on relative water content (RWC) and crop growth rate (CGR) of mung bean under chromium toxicity

**Fig. 1:**
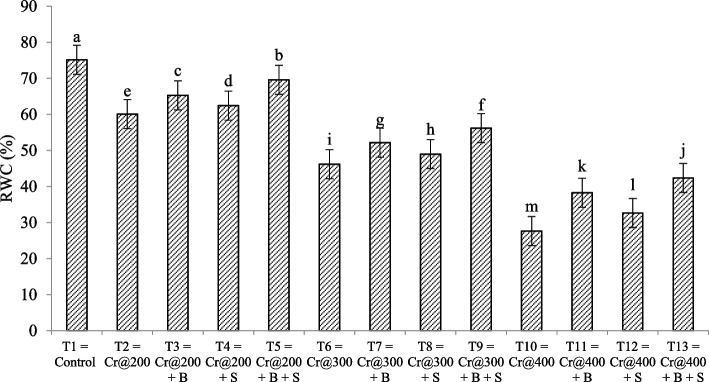
Effect of salicylic acid and *Azospirillum brasilense* on relative water content (RWC) of leaf of mung bean plant under Cadmium stress. Different letters are significantly different in each columns ( *P*  ≤ 0.05)

**Fig. 2:**
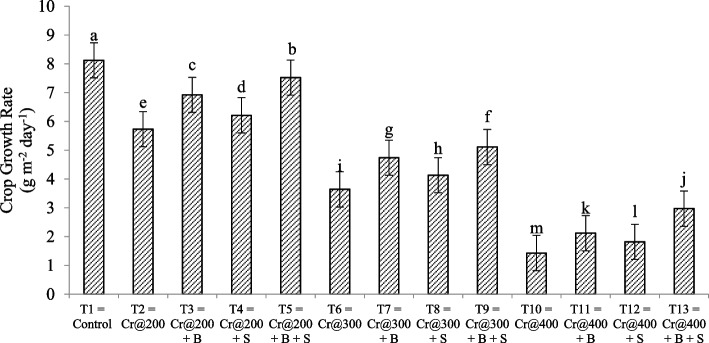
Effect of salicylic acid and *Azospirillum brasilense* on crop growth rate of mung bean plant under Cadmium stress. Different letters are significantly different in each columns ( *P*  ≤ 0.05)

Relative water content (RWC) significantly affected by all studied treatments (Fig. 1). Highest RWC was noticed in T_1_ (75.15%) under control condition followed by T_5_ (69.57%) when Cr toxicity was @200 mg/kg of soil with *A. brasilense* and SA. Lowest RWC were noticed in T_10_ (27.64) when Cr toxicity was @ 400 mg/kg of soil having no soil amendment. Crop growth rate (CGR) also affected by the application of *A. brasilense* and SA under Cr toxicity (Fig. 2). Highest CGR was noticed in T_1_ (8.12 g m^−2^ day^−1^) followed by T_5_ (7.52 g m^−2^ day^−1^) and lowest CGR was observed in T_10_ (1.43 g m^−2^ day^−1^).

## Discussion

Variety of growth parameters in mung bean plants were examined in our study, including plant height, shoot and root length, root-shoot dry and fresh weights, chlorophyll content, enzymatic activities, relative water content (RWC), and crop growth rate. Salicylic acid (SA) and *Azospirillum brasilense* have the significant impact on all studied treatments and can improve the growth under Chromium (Cr) toxicity. Environmental conditions severally effect on the growth and development of the plants [3, 4]. It is difficult for every organisms to grow better under any kind of the stress condition [4, 24]. *A. brasilense* stimulates root development, nutrient uptake, and possibly metal detoxification [25], while SA’s function as a signaling molecule sets off defense responses and antioxidant activities [26, 27]. This coordinated effort maximizes nutrient availability and stress reduction, supporting the growth and resistance of mung bean plants to difficulties brought on by Cr [28, 29]. Chromium (Cr) toxicity blamed for a decrease in growth and yield characteristics by interfering with crucial physiological processes, causing oxidative stress, and competing with necessary nutrients [30]. The Cr toxicity ameliorate by salicylic acid and *A. brasilense* in mung bean growth by activation of defense mechanisms by salicylic acid [15] and the encouragement of root growth and nutrient solubilization by Azospirillum [31]. Salicylic acid primes defense responses while Azospirillum improves nutrient uptake that result in the increase of resilience and productivity in mung bean plant under chromium stress conditions.

### Effect of *Azospirillum brasilense* and salicylic acid on growth related parameters of mung bean under chromium toxicity

A greater reduction of plant vegetative growth parameters was observed in mung bean plants that were exposed to higher Cr concentrations, moving downward with the increase of chromium concentration in the soil. 15.64%, 24.42%, 37.15% decrease in plant height was noticed when Cr toxicity was @200 mg/kg of soil, @300 mg/kg of soil and 400 mg/kg of soil, respectively. But this decrease in percentage remains 6.33%, 14.12%, 27.80% when *A. brasilense* and SA applied in a combine form. Same kind of the trends were observed in root, shoot length and dry weight. A similar pattern of the Cr concentration effect was reported by Husain, et al. [32] about the effect of Cr on the reduced growth of the shoot and root length of the mung bean. Germination and seedling establishment are critical stages in the life cycle of plants and can be affected in the presence of high levels of metals in the contaminated environment [33]. Our findings confirmed that plant growth was affected by different concentrations of Cr and with the higher concentration higher negative effects were noticed. Murtaza et al. [6] observed the same findings under Cr toxicity. Since beneficial microbes are killed off during the contamination process, polluted soils often have low nutrient levels or, in rare cases, nutrient shortages [34]. Metal-tolerant microorganisms, in particular plant growth-promoting rhizobacteria, can be added to such soils to make them nutrient-rich, which not only gives plants thriving in contaminated settings the nutrients they need but also plays a vital role in heavy metal detoxification [31, 34]. Salicylic acid (SA) and *Azospirillum brasilense* enhance a variety of growth metrics under chromium (Cr) toxicity [28, 29]. As a signaling molecule, salicylic acid activates the plant’s protective mechanisms in response to stressors like Cr poisoning. It increases the activity of antioxidant enzymes, minimizing oxidative damage and encouraging development [35]. Soil microbes encourages root growth, improves nutrient and water uptake, and may possibly help with metal detoxification by boosting microbial activity in the rhizosphere [31, 36]. Improved plant height, shoot and root length, as well as root-shoot dry and fresh weight, are the results of the combination application, which maximizes stress protection and nutrient availability while also improving the mung bean plants’ capacity to deal with Cr toxicity.

### Effect of *Azospirillum brasilense* and salicylic acid on chlorophyll contents of mung bean under chromium toxicity

20.24%, 35.64%, 52.09% decreases in the chlorophyll contents were noticed when soil toxicity was @200 mg/kg, 300 mg/kg and 400 mg/kg of soil, respectively. But this chlorophyll reduction due to Cr toxicity remains 3.02%, 23.26%, 38.97% when *A. brasilense* and SA applied in a combine form. Salicylic acid (SA) and *Azospirillum brasilense* increase chlorophyll content under chromium (Cr) toxicity. Initiating defense mechanisms that lessen oxidative stress brought on by Cr toxicity, SA functions as a signaling molecule [35, 37, 38]. As a result, the integrity of the chloroplasts is preserved and chlorophyll breakdown is avoided. *A. brasilense* promotes root development and nutrient uptake, which enhances the physiological health of the plant and increases chlorophyll synthesis [31]. Under Cr stress circumstances, the combined effects of these substances optimize stress adaption, resulting in increased chlorophyll content and therefore better photosynthetic efficiency in mung bean plants [39, 40].

### Effect of *Azospirillum brasilense* and salicylic acid on enzymatic activities of mung bean under chromium toxicity

Salicylic acid (SA) and *Azospirillum brasilense* increase enzymatic activity under chromium (Cr) toxicity. Superoxide dismutase (SOD), peroxidase (POD), and catalase (CAT) are examples of antioxidant enzymes that are stimulated by the signalling molecule SA. These enzymes prevent cellular damage and sustain cellular functions, including enzymatic activities [41], by reducing the oxidative stress brought on by Cr toxicity [40, 42]. Lu et al. [43] reported the production of certain enzymes due to the production of different growth hormones. Azospirillum brasilense improves nutritional status and overall plant health by encouraging root development and nutrient uptake [31]. As a result, the cofactors and substrates required for proper enzyme activity are provided, boosting the antioxidative defense systems. SA and A. brasilense work together to maximise the antioxidative response, allowing for improved enzymatic activities that successfully reduce Cr-induced oxidative stress and promote mung bean growth [28, 29].

### Effect of *Azospirillum brasilense* and salicylic acid on relative water content (RWC) and crop growth rate (CGR) of mung bean under chromium toxicity

Salicylic acid (SA) and *Azospirillum brasilense* increase relative water content (RWC) and crop growth rate (CGR) under chromium (Cr) toxicity. SA improves the growth hormones production in the plant that improves the physiological parameter of mung bean. 20.08%, 38.53%, 63.22% decrease in the RWC was observed when Cr toxicity was@ 200 mg/kg, 300 mg/kg and 400 mg/kg of soil, respectively. But this decrease in RWC was remains 7.42%, 25.22%, 43.63% when *A. brasilense* and SA applied in a combine form. 29.43%, 55.17%, 82.38% decrease in the CGR was noticed under Cr toxicity of 200 mg/kg soil, 300 mg/kg of soil and 400 mg/kg of soil, respectively. But this reduction remains 7.38%, 37.06%, and 63.42%, respectively when *A. brasilense* and SA applied in a combine form. The signaling molecule SA triggers the expression of genes that control water management in response to stress [44, 45]. It enhances stomatal control, which lowers transpiration water loss and keeps RWC greater [46]. As a result of *Azospirillum brasilense* promotion of root growth, plants are better able to reach water in deeper soil layers, which improves overall water intake and leads to enhanced RWC [31]. Plants are able to better utilize water resources for growth and stress adaption thanks to the increased nutrient availability brought on by Azospirillum inoculation, thus boosting RWC and CGR. SA and A. brasilense work together to maximize water use, improving both RWC and CGR and enabling mung bean plants [28, 29].

## Conclusion

Chromium stress has a negative effect on growth and productivity of mung bean but seed inoculation of *Azospirillum brasilense* and foliar application of salicylic acid together mitigate the adverse effect of Cr stress. Higher Cr toxicity has the higher negative effect. Sole application of A. brasilense and SA is effective to improve the mung bean growth but it is not enough to control negative effect of Cr toxicity. Combine application of *A. brasilense* and SA is more effective to mitigate the negative effect of Cr toxicity. Use of *A. brasilense* and SA is very effective for sustainable crop production even under adverse environmental conditions. In future, there is need to investigate and make an approach with the use of A. brasilense and SA by which farmers can get fully control on Cr toxicity.

## Data Availability

The datasets used and/or analysed during the current study available from the corresponding author on reasonable request.
